# The World Health Organization guideline for non-surgical management of chronic primary low back pain in adults: implications for equitable care and strengthening health systems globally

**DOI:** 10.1186/s41256-025-00426-w

**Published:** 2025-07-07

**Authors:** Andrew M. Briggs, Yuka Sumi, Anshu Banerjee

**Affiliations:** 1 World Health Organization, Geneva, Switzerland; 2 Faculty of Health Sciences, Curtin School of Allied Health, Curtin University, GPO Box U1987, Perth, WA 6845 Australia; 3 Department of Maternal, Newborn, Child and Adolescent Health and Ageing, World Health Organization, Geneva, Switzerland

**Keywords:** Chronic pain, Health policy, Health systems, Universal Health Coverage, Musculoskeletal

## Abstract

The World Health Organization (WHO) released its first guideline on non-surgical management of chronic primary low back pain (LBP) in adults in 2023. The Guideline signals a timely and important global response to the current and projected burden attributable to LBP, particularly amongst populations made vulnerable, e.g. older people and people in low- and middle-income countries. The Guideline offers recommendations on which interventions for chronic primary LBP should be offered in most contexts, and those interventions that should not be offered as part of routine care. As a WHO technical product, the Guideline can function as a catalyst to commence reorientation of health systems and service delivery towards holistic, integrated, person-centred and high-value LBP care. However, to achieve this vision and Universal Health Coverage that is inclusive of LBP care, transformations are required at point of care (micro-level), within service models and organisations (meso-level) and across health system Building Blocks (macro-level). In this paper, we consider implications of the Guideline for health services and health systems globally. We highlight opportunities and priorities to build capacity across the Building Blocks of health systems and how current global health agendas, such as the WHO 14th General Program of Work, UN Decade of Healthy Ageing 2021–2030, and Rehabilitation 2030 can support implementation of the Guideline’s recommendations. Global collaborative and multi-sectoral efforts, supported by countries, health workers, professional/academic societies, health financing agencies, civil society organisations, private sectors, and people with lived experience will be needed to sustainably implement the Guideline’s recommendations into health systems globally.

**Supplementary Information:**

The online version contains supplementary material available at 10.1186/s41256-025-00426-w.

## Introduction

The World Health Organization (WHO) launched its first clinical guideline on non-surgical management of chronic primary low back pain (LBP) in adults in primary andcommunity care settings (https://www.who.int/publications/i/item/9789240081789) in December 2023 (https://www.who.int/news/item/07-12-2023-who-releases-guidelines-on-chronic-low-back-pain) [1]. Chronic primary LBP (ICD-11 code MG30.02) (https://icd.who.int/browse/2024-01/mms/en#1236923870) refers to a persistent or recurrent pain experience of more than three months duration that is not reliably attributed to an underlying disease process or structural lesion—estimated to account for at least 90% of chronic LBP presentations in primary care [2]. More than 38,000 downloads of the Guideline were recorded in the first 6 months after its publication. As a WHO Public Health Goods Technical Product (https://www.who.int/our-work/technical-products), the Guideline supports activities aligned to the goals of Agenda 2030, specifically, accelerating towards Universal Health Coverage (UHC) and better health and wellbeing. With primary and community care as the intended setting for the Guideline, it serves to strengthen primary care systems, recognised as the most efficient mechanism to improve health equity and health and social wellbeing [3]. Importantly, the Guideline extends and builds on other WHO technical products that aim to: i) improve functioning and wellbeing for people with chronic pain, ii) support healthy ageing through integrated care, and iii) strengthen rehabilitation capacity in health systems. The Guideline focuses on management of chronic primary LBP in adults. Other LBP presentations such as acute symptoms and chronic secondary LBP were not considered. Assessment, diagnosis and care pathways were not considered within the scope of the Guideline. Rather, these topics may reasonably be considered as subsidiary products to support implementation efforts within countries.

The purpose of this article is to provide an overview of the Guideline, position implementation opportunities in the context of current global health agendas and WHO programs, and highlight implications for health systems and service delivery. We frame implications for health systems and service delivery as globally-relevant considerations and opportunities for improving service delivery for adults experiencing chronic primary LBP and for health system performance. Individual countries may consider these against local context to determine a locally-relevant reform agenda to support implementation of the Guideline. The intended audience is health services and systems managers and stakeholders working in global public health.

### Low back pain matters to people and health systems

For some patients, the experience of LBP is disrupting but transient, while for others, pain persists and affects all areas of their lives, impacting their quality of life and self-identities [4, 5]. While the experience and sequelae of pain are highly individual, chronic symptoms are typically associated with reduction in physical and mental capacities (i.e. intrinsic capacity) and functional ability, including social participation. Evidence from inception cohort studies suggests that most people with an episode of acute LBP experience recovery from pain and disability within the first 6 weeks. However, a proportion of people continue to experience symptoms beyond 12 weeks [6] with various trajectories, including recurrent episodes [7, 8], which contribute to the burden of chronic LBP. Older people may also experience less favourable trajectories of pain and disability, often with recurrent episodes [9]. Importantly, people experiencing chronic symptoms are a distinct group. Among inception cohorts with chronic symptoms (12 to < 52 weeks), variable pain and disability trajectories that persist over time are observed [6].

The most recent 2020 estimates from the Global Burden of Disease (GBD) study continue to identify LBP as the leading cause of disability globally, accounting for 8% of all years lived with disability (YLDs), affecting almost 619 million people—about 1 in 13–a 60% increase in cases since 1990 [10], with a disproportionate burden experienced by females and older people. For example, global prevalence rates of LBP are higher in females compared with males, with this sex-based differential becoming more pronounced later in life (age > 75 years). Even when age-standardised, a disproportionately higher prevalence was estimated in females[10]. Over time, the steepest rise in disability was estimated for the low and middle-income countries (LMICs), attributed to rapid population ageing and expansion, particularly in African countries and countries of Oceania. Although the age-standardised prevalence is decreasing, current projections estimate that by 2050, the total absolute number of LBP cases is expected to increase by 36% from 2020 estimates, to 843 million people[10], signalling the need to strengthen health systems and consider LBP within a broader healthy ageing agenda [11]. Nonetheless, GBD estimates for LBP should be interpreted with caution given that many estimates rely on modelling due to an absence of primary data, especially from the LMICs [12], and model assumptions may not be valid across different settings, such as a uniform severity distribution [13].

While LBP is experienced across the lifecourse from late childhood, prevalence peaks in older age [10], and older people more commonly experience incapacitating and persisting LBP symptoms [14], which substantially impacts their intrinsic capacity and functional ability [15]. Older people with chronic LBP are more likely to experience comorbidities compared to older people without LBP, such as falls [16], depressive symptoms [17] and poor sleep [18], while older people with more severe musculoskeletal pain are more likely to experience frailty [19].

Coupled with the health burden, the financial and human capital cost of LBP to individuals, health systems and society appears to be substantial and increasing in high-income and low-income settings where these few data are available [20, 21]. Therefore, technical guidance on management of chronic LBP and guidance that explicitly considers the health and wellbeing of older people is important and relevant to countries and the UN Decade of Healthy Ageing 2021–2030. Despite the disproportionate burden of LBP experienced by older people [14, 15], there is a lower volume of LBP research published on older people compared to people of working-age [22]. Further, other systematic reviews have identified that older adults are frequently under-represented through exclusion in clinical trials for LBP [23–25]. Collectively, this results in less guidance available to clinical practice and health system improvements for older people.

There are substantial opportunities to improve LBP care and LBP-related health equity. Currently, care for LBP is frequently at odds with contemporary evidence, creating a ‘low-value’ care landscape characterised by diverse and potentially harmful medications, inappropriate imaging,  invasive procedures, and unhelpful non-pharmacologic interventions [26, 27]. Care approaches are inadequately person-centred and inequities in health outcomes persist for groups rendered vulnerable [27, 28]. Disparities in chronic pain care are common in high-income countries, driven by ethnicity and socioeconomic status [29, 30], while delivery of interventions for LBP in LMICs is frequently discordant with current guidelines [31] and care priorities expressed by people who live with chronic pain [32]. Musculoskeletal conditions, such as LBP, frequently co-exist with other noncommunicable diseases (NCDs)[33–35]. Improving care for LBP has the potential to improve health gains related to other NCDs by enabling people to better participate in rehabilitation and re-engage in social participation, such as work [36, 37].

## WHO Guideline recommendations and their implications for care (micro-level) and health services (meso-level)

**Fig. 1:**
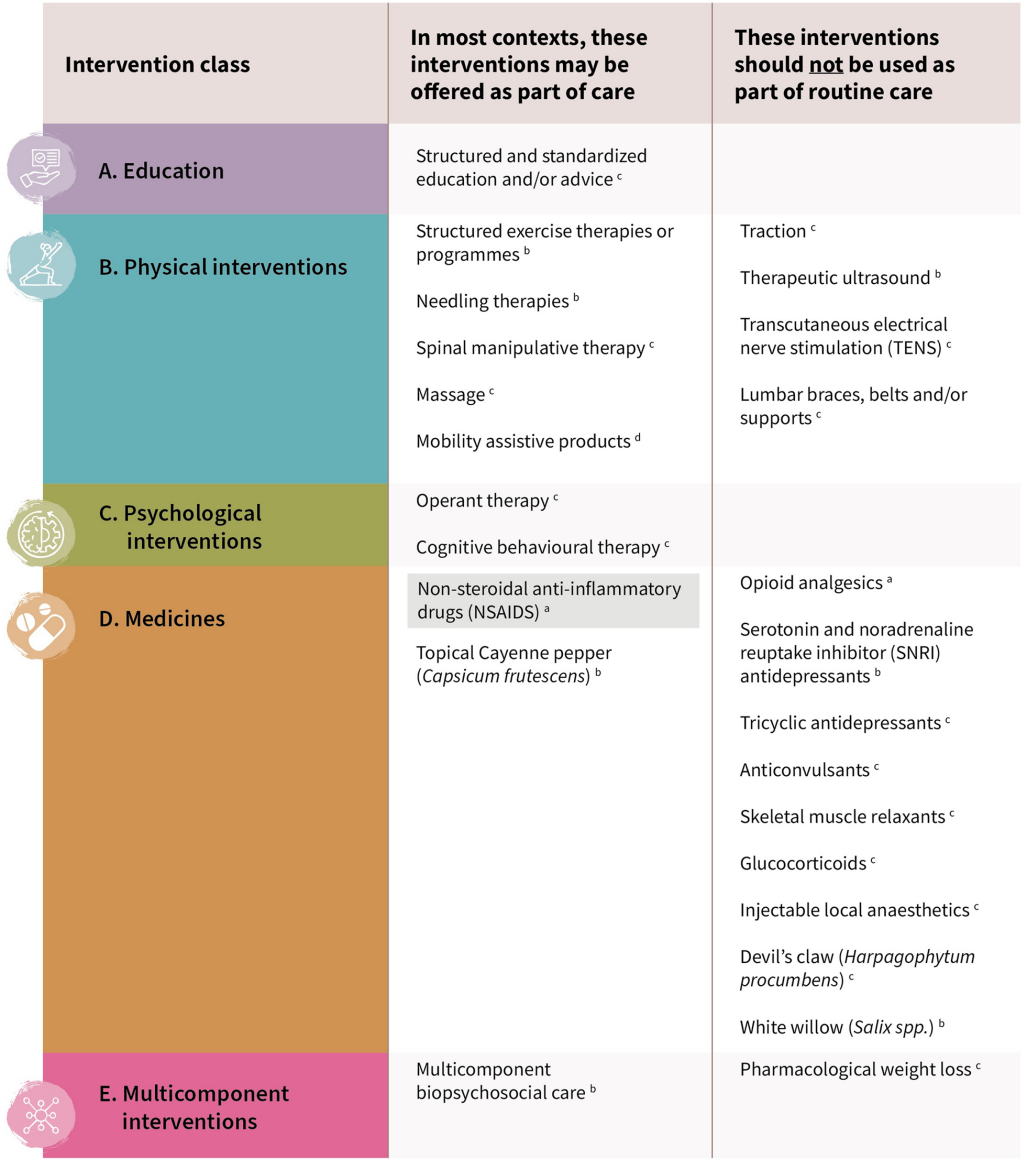
Summary of recommendations of interventions that may be offered as part of care in most contexts, and interventions that should not be used as part of routine care in adults experiencing chronic primary LBP, with or without spine-related leg pain, in community and primary care settings. All recommendations are relevant to older people, other than non-steroidal anti-inflammatory drugs (NSAIDs)—the recommendation in favour of using NSAIDs does not extend to older people. Interventions are organised by class and superscripts indicate the judgements of the certainty of the evidence made by the Guideline Development Group. No recommendations were made for 12 interventions, including: respondent therapy, cognitive therapy, mindfulness-based stress reduction therapy, paracetamol (acetaminophen), benzodiazepines, cannabis-related pharmaceutical preparations for therapeutic use, topical Brazilian arnica, ginger, topical white lily, topical combination herbal compress, topical combination transdermal diffusional patch, and non-pharmacological weight loss. Figure reproduced from WHO (https://www.who.int/publications/i/item/9789240081789) under a Creative Commons Attribution-NonCommercial-ShareAlike 3.0 IGO licence (CC BY-NC-SA 3.0 IGO).^a^: moderate certainty evidence. ^b^: low certainty evidence. ^c^: very low certainty evidence. ^d^: good practice statement

Developed according to WHO standards [38], the Guideline considers 37 non-surgical interventions deemed feasible for delivery in primary and community care settings globally, organised by 5 classes: education, physical, psychological, medicines and multi-component interventions. Informed by systematic reviews of randomised controlled trials (RCTs) published to mid-2022, the Guideline offers 24 recommendations and one good practice statement. No recommendations were made for 12 interventions due to either inadequate or absent evidence, or where the balance between benefits and harms was equivocal (Fig. 1). Among the 367 included RCTs, 193 were conducted in 26 high-income countries, 72 in 5 upper-middle-income countries, 72 in 6 lower-middle-income countries, and 30 were multi-national trials.

Across interventions, the overall judgements on the certainty of evidence ranged from moderate to very low, resulting in all recommendations formulated as ‘conditional’ in strength (Fig. 1). For clinical practice, this infers that the interventions recommended ‘*in favour*’ should be made available and offered as part of care to adults experiencing chronic primary LBP in most contexts, while those recommended ‘*against*’ should not be offered as part of routine care. The strength of these recommendations is not surprising since the evidence for benefit in most interventions tested in isolation in RCTs was modest at best. This is consistent with earlier [39, 40] and more recent reviews [41], and considering that chronic primary LBP has a multidimensional aetiology, it is intuitive that unimodal interventions delivered in isolation are less likely to confer substantial benefit. Further, outcomes were generally limited to short- and intermediate-term follow-up; outcomes disaggregated for older people were inconsistently reported; and for most interventions, the RCTs were more frequently conducted in high-income countries. Given the conditionality of the recommendations, clinical judgement and shared-decision making are important to informing intervention selection.

Although the recommendations are largely consistent with earlier guidelines for management of LBP in primary care settings [42], the WHO Guideline has unique and important implications for practice. First, it is the only guideline for chronic primary LBP that offers globally-applicable recommendations that are adaptable to local context with specific consideration of benefits and harms of interventions and other considerations for older people [43]. Second, the Guideline provides a contemporary snapshot of evidence of benefits and harms of interventions anchored to consistent inclusion criteria, comparators, outcomes and quality appraisal and certainty assessment methods. This is important because although many systematic reviews of common interventions for LBP are available [44], health workers are faced with the complexity of interpreting disparate evidence syntheses (and ultimately guidelines) with variable inclusion criteria, comparators and appraisal methods.

**Fig. 2:**
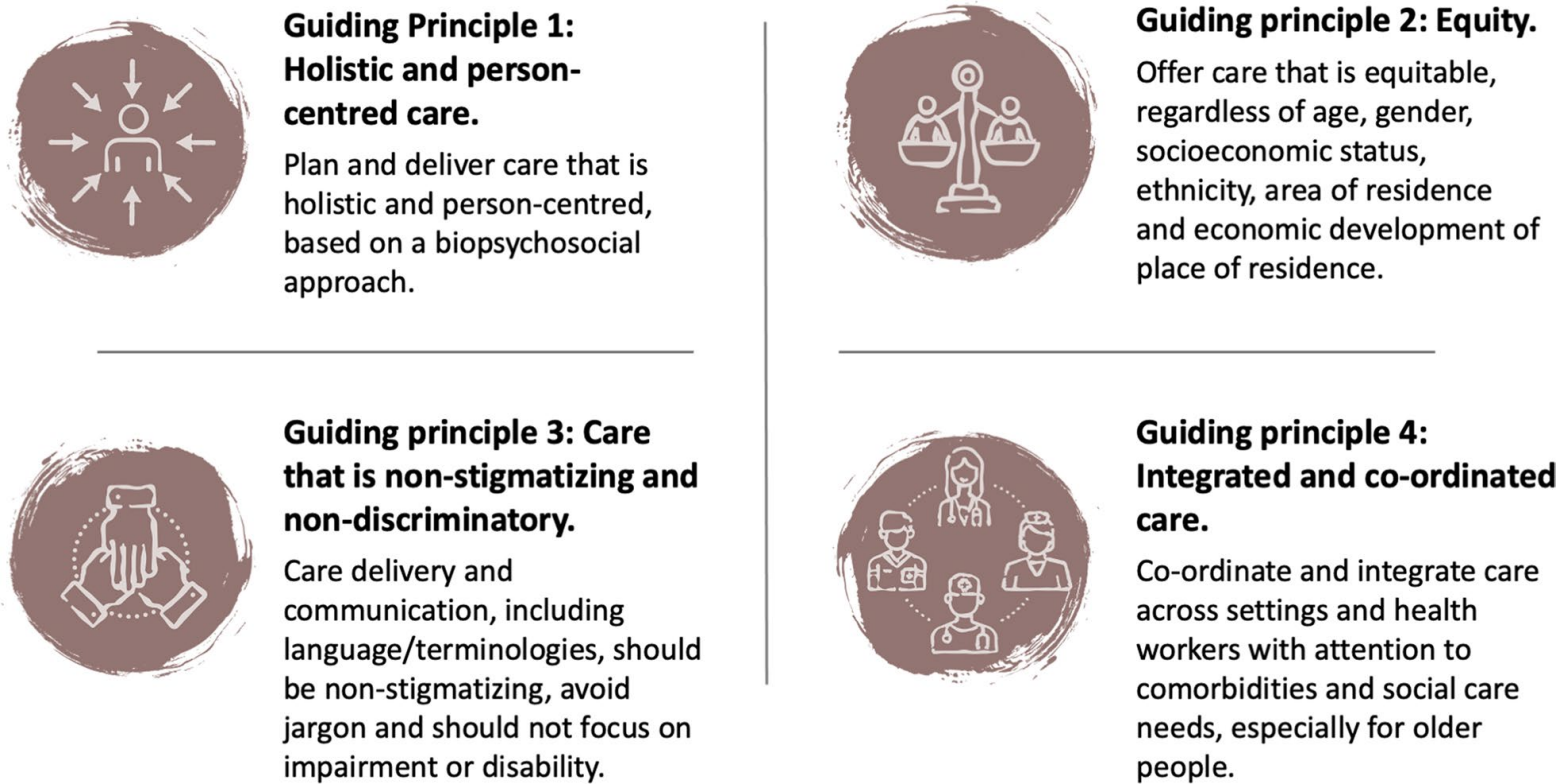
Guiding principles for the management of chronic primary LBP in adults, reproduced from WHO (https://www.who.int/publications/i/item/9789240081789) under a Creative Commons Attribution-NonCommercial-ShareAlike 3.0 IGO licence (CC BY-NC-SA 3.0 IGO)

As the understanding of the biology and experience of chronic pain has evolved [45], so too has the approach to chronic pain care, where holistic assessment and personalised (tailored) care planning and delivery from a biopsychosocial perspective is now widely advocated, and valued and expected by patients [43, 45]. In practice, this means that a suite of interventions may be appropriate for an individual experiencing chronic primary LBP, provided in a tailored package that targets the combination of factors contributing to that person’s pain experience. RCTs that evaluate this approach are lacking [46], creating a lag between practice and evidence. Nonetheless, some promising evidence is now emerging [47]. Within the Guideline, other than multicomponent biopsychosocial care, all other interventions were evaluated as single, stand-alone interventions against a comparator. This gap between integrated person-centred care for chronic musculoskeletal pain and current RCT evidence is one of the most important for patients, health workers and health services. Such evidence is needed to build workforce competencies and strengthen service models and systems to deliver holistic, person-centred and integrated LBP care. Responding to limitations in the current evidence landscape, the Guideline offers practice guidance through Guiding Principles that underpin all interventions and approaches to care (Fig. 2), and Clinical Practice Considerations relevant across interventions (Supplementary file 1). Indeed, the Guiding Principles are relevant to care of all health conditions, and if adopted at scale, have the potential to improve holistic, person-centred care and health equity through supporting timely and accessible care.

## Building and sustaining a global public health response to chronic primary LBP: implications for health systems (macro-level).

While the WHO Guideline is a timely initial step in a global public health response to chronic primary LBP (i.e. a response that includes organised efforts to improve health and wellbeing outcomes), it cannot be the single ‘silver bullet’ to solve this complex health issue. Nonetheless, technical guidance offered by WHO can catalyse strengthening of health systems to improve health and wellbeing and accelerate towards UHC [48]. WHO technical products signal the global health importance of a health topic, thereby raising its priority within the global health agenda; support health system strengthening efforts within countries [48]; and provide an entry point for a pipeline of products and tools to support implementation efforts. WHO technical products also integrate with other global health programmatic activities and priorities, such as WHO’s General Program of Work (GPW), the Sustainable Development Goal (SDG) 3.8 target for UHC, and relevant resolutions such as the UN Decade of HealthyAgeing (2021–2030) (https://www.who.int/initiatives/decade-of-healthy-ageing) and Rehabilitation 2030 (https://www.who.int/initiatives/rehabilitation-2030). Supplementary file 2 outlines the opportunities to strengthen capability in LBP care within health systems through integration of LBP care responses with these strategic global health programs and priorities. Similarly, integrating global Public Health Goods, such as the Guideline, within the broader GPW and Agenda 2030 targets can serve to strengthen efforts in achieving the SDG target for UHC.

Transitioning from global technical or strategy guidance to national and/or sub-national implementation is critical for improving health and wellbeing outcomes [49, 50]. While WHO can provide leadership, technical guidance and implementation guidance, countries need to adopt the recommendations according to local context and priorities and determine national-level implementation actions. Building capacity within countries to deliver holistic, person-centred and integrated LBP care within national health systems may require strengthening across several areas [51, 52]. Theoretic systems strengthening frameworks provide guidance around the breadth of priority areas that could be addressed [53]. While various health systems strengthening or policy capacity frameworks may be applicable in this context [54, 55], the *WHO Health Systems Building Blocks* model is the most widely recognised and adopted [56], and the foundation on which newer frameworks have evolved [55]. We acknowledge that the World Bank/Harvard “*Control Knobs*” framework and recent models that are musculoskeletal-specific may also be fit for purpose [51, 57]. The WHO Health Systems Building Blocks model is intended to provide a simple and common language for deriving and organising actions for systems strengthening. However, it does not account for dynamic inter-relationships between Building Blocks and actions that might be critical outside these foci [56], such as integration between health and social care systems, community empowerment and harnessing lived experience to drive person-centred care [28, 58]. In this context the Building Blocks framework can be used to highlight and guide potential areas of action, while local expertise and local context should inform how strengthening efforts should be operationalised.

### Building block 1: leadership and governance

Relative to other health conditions, musculoskeletal conditions such as LBP are often not prioritised at a level commensurate with their population health burden. Evidence of relatively lower prioritisation and leadership by national governments can be seen in policy, where national policies or priorities for musculoskeletal health are lacking or nascent [51, 59]. This burden-policy response mismatch partly reflects that global health performance targets for NCDs are linked to reducing premature mortality, rather than (explicitly) disability, and that policy foci in LMICs reflect other health priorities, such as communicable diseases and maternal and newborn health. For countries to act on LBP care, health policies will need to evolve to include musculoskeletal health as a priority, with LBP as a key feature. Raising the policy priority for musculoskeletal health also requires greater awareness, leadership and prioritisation within communities and a commitment from governments to integrate lived experience into policy formulation [28, 60]. For example, WHO provides guidance to Members States in meaningful engagement of people living with NCDs and older people to co-create and enhance policies, programmes and services relevant to their health [11, 60]. Engaging, empowering and educating communities across sectors to participate in the health debate about musculoskeletal health is recognised as a key action to elevate its priority status [51]. Here, an opportunity exists to integrate helpful public health messaging about LBP care with other public health campaigns such as healthy ageing, physical activity, rehabilitation and prevention and control of NCDs.

### Building block 2: service delivery

Translating recommendations from the Guideline and other technical products to practice within countries will require development of local service models and pathways, including care standards and indicators, such as those developed recently in Australia [61]. Locally-acceptable, feasible and co-created service models/pathways for musculoskeletal conditions, such as LBP care, integrated with service delivery for other health conditions or services will be important to deliver accessible, acceptable and sustainable care, especially in LMICs. For example, LBP care in older people has recently been integrated into the WHO Integrated Care for Older People (ICOPE) approach within pathways to address mobility loss [62]. While a person-centred approach should be applied across care pathways, how each pathway is operationalised may differ according to the local health system, infrastructure, resourcing and workforce capacity. For example, different service models for LBP care have been proposed, including triage-based/risk stratified care, phenotype-based care, and stepped care; yet there is insufficient evidence that one approach is superior to another across settings [63], highlighting the need for implementation efforts that are context- and/or setting-specific.

The Guideline’s recommendation in favour of biopsychosocial care delivered by a multidisciplinary team is particularly relevant to implementation feasibility across settings. How this intervention could be implemented in different countries will necessarily differ. In some high-income settings, a requisite workforce, financing, referral pathways and service models may be available to deliver multidisciplinary biopsychosocial care (e.g. in a pain clinic) where indicated, while in other settings such as rural and remote areas or low-resource settings, this intervention may be unfeasible or delivered differently.

### Building block 3: health system financing

The interventions recommended to be offered to adults with chronic primary LBP are intended to be made available within countries as a suite. Within this approach, health workers can better tailor care by selecting and sequencing interventions according to the person’s needs, based on a biopsychosocial assessment. In some countries, ensuring availability and affordability of interventions may require transformation to regulatory frameworks and financing mechanisms, including roles and responsibilities of health workers. WHO resources such as the UHC Service Planning, Delivery and Implementation (https://uhcc.who.int/uhcpackages/) (SDPI) platform and Rehabilitation in health (https://iris.who.int/handle/10665/375712) financing guide can serve as enablers to these transformations by providing guidance to countries on interventions, and human and material resources needed for effective service delivery packages that could be made available without financial burden. In some contexts, de-implementation and de-funding of interventions that should not be offered as part of routine care may reorient financing towards those interventions that should be offered.

### Building block 4: health workforce

While workforce stock and distribution need addressing across all cadres and for all conditions, additional efforts are needed to close competency gaps for workforce cadres that provide services to people with chronic musculoskeletal pain. Here, building capacity to provide chronic pain care within a person-centred and biopsychosocial approach is needed, as many cadres lack capabilities in this approach [64]. Supporting competency development in chronic pain care needs to be coupled with support to discontinue practices that are not recommended in most contexts, such as routine use of some pain medicines or electrotherapies. Resources, tools, pre-licensure and post-graduate training and, in some cases, service or financial incentives, may be required to support workforce to deliver recommended care [64]. The development of diagnostic, triage, referral and care pathways, care standards, and transdisciplinary competency standards in LBP care may be helpful to build workforce capacity [65, 66]. Although diverse disciplines provide care to people who experience musculoskeletal pain, which can create complexity in care pathways, fragmented care provision and challenges to integrated care, this context also creates the opportunity to build capacity in LBP care at scale across disciplines. This highlights the need to adopt a transdisciplinary approach to capacity-building efforts based on a common clinical reasoning framework and shared understanding for care [66]. Resources to support health workers deliver care aligned with the recommendations and Guiding Principles in the Guideline are summarised in Supplementary file 3.

### Building block 5: medical products, vaccines and health technologies

Among the 19 medicines evaluated in the Guideline (including cannabinoids and herbal medicines), only non-steroidal anti-inflammatory drugs (NSAIDs) and topical cayenne pepper were recommended to be offered in most contexts, since these agents are typically widely available. However, the recommendation for NSAIDs does not apply to older people due to the lack of evidence for benefits and evidence for potential harms. On the other hand, there is global disparity in access to assistive technologies, with as few as 3% of people in some LMICs being able to access the assistive technologies they need, compared with 90% in some high-income countries [67]. Older people with LBP and spine-related leg pain are more likely to require an assistive product for walking, compared to older people without LBP [16]. The Guideline provides a good practice statement indicating that quality, affordable mobility assistive products should be offered, when indicated through a person-centred assessment. The *WHO and UNICEF Global report on assistive technology* provides recommendations on how countries can enhance access to assistive technologies [67].

### Building block 6: health information systems

Population health conditions surveillance should be integrated across health conditions/health states to enable accurate and holistic population health data. In many countries, however, population health surveys do not collect information on prevalence or impact of musculoskeletal conditions [68], evidenced by a lack of primary prevalence data for LBP in GBD studies [12]. Nonetheless, experience from the Solomon Islands, for example, identifies that integrating musculoskeletal items into existing national health surveillance is feasible [69]. Building capacity within national health surveillance infrastructure and processes to collect data on national prevalence, disability and functioning data related to musculoskeletal conditions, particularly LBP, will be important to inform planning related to service need and equity, workforce requirements and health expenditure [12, 13]. For example, indicators for LBP experience (prevalence, impact and outcomes) and care (service requirements and coverage) could be integrated into monitoring and evaluation systems for the Measurement, Monitoring and Evaluation of the UN Decade of HealthyAgeing (https://www.who.int/groups/technical-advisory-group-for-measurement-monitoring-and-evaluation-of-the-un-decade-of-healthy-ageing), Rehabilitation (https://iris.who.int/handle/10665/354390), and NCDs (https://www.who.int/teams/ncds/surveillance/monitoring-capacity/gmf). Importantly, disaggregation of data by age, gender and setting will support service development for community segments in most need [51]. Comparing data between countries requires consensus definitions be established and used for LBP in health information management systems that are consistent with ICD-11 classifications, as well as using standardised definitions and outcomes for LBP in population health surveillance, such as those already established [70, 71].

## Implications

The WHO Guideline represents an important and anticipated starting point for a global public health response to chronic primary LBP. System (macro)-, service (meso)- and clinical (micro)-level transformations to support care delivery aligned with the Guideline’s recommendations are likely to redress, in part, the current landscape of low value care for LBP and contribute to reducing its burden. Transitioning from technical guidance to implementation in countries and realising health and wellbeing benefits for people will require dedicated and sustained efforts and resourcing from multiple levels and areas of health systems, spanning each of the Building Blocks, and from healthcare workers, people living with LBP and other stakeholders. It will be important to maintain dissemination and implementation efforts and monitor and share implementation experiences between countries. Key opportunities to strengthen health systems to deliver equitable, person-centred care for chronic primary LBP include:

- Evolve national health policies to include musculoskeletal health (with LBP as a key focus) and empower and educate communities on musculoskeletal wellbeing and care.
- Co-create local models of care and service models for LBP that integrate with existing pathways and infrastructure. Determine how a multidisciplinary approach could be applied and realized to improve health and wellbeing for some people living with chronic primary LBP.
- Work towards making recommended interventions available to health workers to select and sequence according to a person’s needs, without imposing financial burden to people, including assistive products.
- Build transdisciplinary workforce competencies and capabilities in chronic pain care.
- Integrate indicators to collect experiences and outcomes for chronic LBP care into national health surveillance systems using standardised definitions.

Limitations in the certainty of the evidence informing the Guideline may limit the potential health and wellbeing benefits. The certainty of the available evidence from RCTs was generally low to very low for most interventions, largely ascribed to a lack of high-quality RCTs. Therefore, undertaking more low-quality RCTs will not be helpful to clinical decision-making or to strengthen service delivery and systems for LBP care, particularly for behavioural interventions. While high-quality RCTs of interventions compared to placebo, no care or usual care are critical for judging benefits (and to some extent, harms) of interventions, such evidence must extend to under-represented and groups rendered vulnerable, such as older people, individuals living with comorbidities, people experiencing intersectoral disadvantage and people in vulnerable settings. Further, the translation of trial evidence to guidelines and practice must be supported by other research disciplines, particularly implementation research, health economics research, observational (registry-based) research for monitoring health outcomes and harms in real world settings, and qualitative research that explores the acceptability and feasibility of care approaches. Compared with other NCDs, research funding and capacity in musculoskeletal health is disproportionate to burden of disease, suggesting that greater national investments in musculoskeletal health research is warranted to advance the certainty of evidence of interventions that are high priority for patients, clinicians and health services.

## Supplementary Information

Additional file 1.

Additional file 2.

Additional file 3.

## Declarations

### AI and AI-assisted technologies

No AI was used in the preparation of this manuscript.

## Glossary of terms

**Functioning**: An umbrella rehabilitation term for body functions, body structures, activities and participation. The level of functioning of an individual is the result of the interaction between a health condition and contextual factors (environmental and personal factors).
**Functional ability**: Health related attributes that enable people to be and to do what they have reason to value (meet basic needs, learn, grow, and make decisions, be mobile, build and maintain relationships, contribute).
**High-value care**: Care for which evidence suggests it confers benefit to patients, or probability of benefit exceeds probable harm.
**Intrinsic capacity**: Comprises all the mental and physical capacities that a person can draw on and includes their ability to walk, think, see, hear and remember. The level of intrinsic capacity is influenced by several factors such as the presence of diseases, injuries and age-related changes.
**Low-value care**: Delivery of care or interventions where evidence suggests they confer no or very little benefit on patients, or risk of harm exceeds likely benefit, or, more broadly, the added costs of the intervention do not provide proportional added benefits [72].
**Universal Health Coverage (UHC)**: Means that all people have access to the full range of quality health services they need, when and where they need them, without financial hardship. It covers the full continuum of essential health services, from health promotion to prevention, treatment, rehabilitation and palliative care.
