# Supplementary material for: The World Health Organization guideline for non-surgical management of chronic primary low back pain in adults: implications for equitable care and strengthening health systems globally
Source: Glob Health Res Policy. 2025 Jul 7;10:26. doi: 10.1186/s41256-025-00426-w (PMC12232859; doi:10.1186/s41256-025-00426-w)
Supplement: Supplementary file 1 — Additional file 1. [file 41256_2025_426_MOESM1_ESM.docx]

## Clinical practice considerations offered in the WHO Guideline*(1)*.

1. **Clinical assessment and timely referral.**
   Conduct a thorough clinical assessment from a biopsychosocial perspective by health worker(s) with requisite knowledge and skills to identify which interventions might be appropriate and when, and where further detailed or urgent clinical review/referral may be indicated.
2. **Personalized information and advice.**
   Provide accurate information about chronic primary LBP and personalized advice. Personalized advice means helping people to make sense of their pain experience from a biopsychosocial perspective and support their re-engagement in meaningful life activities.
3. **Tailor a package of interventions.**
   Adults with chronic primary LBP may require a number of interventions to experience benefit, rather than a single intervention delivered in isolation. The selection and sequencing of interventions should: i) address the range of factors that contribute to that person’s LBP experience (physical, psychological, and/or social); and ii) address the person’s context and their needs, values and preferences. Generally, start with the least invasive and least potentially harmful intervention(s).

1. World Health Organization. WHO guideline for non-surgical management of chronic primary low back pain in adults in primary and community care settings. Geneva: WHO; 2023 (<https://www.who.int/publications/i/item/9789240081789>.
