# Supplementary material for: The World Health Organization guideline for non-surgical management of chronic primary low back pain in adults: implications for equitable care and strengthening health systems globally
Source: Glob Health Res Policy. 2025 Jul 7;10:26. doi: 10.1186/s41256-025-00426-w (PMC12232859; doi:10.1186/s41256-025-00426-w)
Supplement: Supplementary file 2 — Additional file 2. [file 41256_2025_426_MOESM2_ESM.docx]

## Opportunities to strengthen capability for LBP care within health systems through integration with current global health priority programs.

**WHO General Program of Work.**

The WHO [14^th^ GPW 2025-2028](https://www.who.int/about/general-programme-of-work/fourteenth) has a focus on delivering global goods for public health through technical products and leveraging such technical products to generate policy options and guidance for countries. This focus is important since many LMICs lack capacity in policy, funding, workforce capacity and healthcare infrastructure to support implementation of WHO guidelines. Strategic outcomes of the 14^th^ GPW to strengthen primary care and accelerate towards UHC are relevant to supporting implementation of the Guideline. Strengthening primary health care is the foundation for UHC, since primary health care is most inclusive, equitable, cost-effective and efficient approach to enhance people’s physical and mental health, as well as social well-being. Since LBP is a common reason for seeking care in primary and community care settings and that non-surgical interventions can be delivered in these settings, LBP care is highly relevant to achieving UHC. The resourcing, infrastructure, systems and support from Member States to drive the GPW priorities and activities to achieve UHC provide an opportunity to disseminate and implement the recommendations in the Guideline at scale.

**United Nations Decade of Healthy Ageing 2021-2030**

The number of people aged ≥ 60 years worldwide will increase from 1.1 billion in 2020 to 1.4 billion by 2030*(1)*, coinciding with an estimated increase in the absolute number of older people experiencing chronic LBP*(2)*. Yet, too little attention has been given to how older people live, the contributions they can make and the health and care services and support they may require. The vision of UN Decade of Healthy Ageing 2021-2030 (The Decade), adopted by World Health Assembly (WHA) and UN General Assembly in 2020, aligned with the Sustainable Development Goals, and implemented through global collaboration, is a world in which all people live long and healthy lives, and to improve the lives of older people, their families and the communities*(3)*. The Decade builds on WHO’s framework for healthy ageing, shifting from disease-based approaches, to enable older people to do things that are important to them in the places where they want to live. Given chronic LBP is more common among older people, actions underpinning the Decade action areas can facilitate implementation of the recommendations in the Guideline and, ultimately may support improved care for older people experiencing chronic primary LBP, especially in the context of integrated primary care services.

**Rehabilitation 2030**

In 2019, some 2.4 billion people were estimated to live with health conditions associated with limitations in their functioning and where benefits could be derived from rehabilitation services; a 63% increase since 1990*(4)*. Among these, around 1.7 billion people lived with a musculoskeletal condition, with LBP being the leading condition likely to benefit from rehabilitation across 134 of the 204 countries evaluated*(4)*. Launched in 2017, the WHO Rehabilitation 2030 initiative aims to promote universal access to rehabilitation, as part of UHC, in response to the substantial unmet need for rehabilitation services globally. The need to expand and integrate rehabilitation services as part of UHC was reinforced in a resolution at the WHA in 2023. Within the WHO Rehabilitation 2030 initiative, the development of technical guidance and resources such as the [Package of Interventions for Rehabilitation](https://www.who.int/activities/integrating-rehabilitation-into-health-systems/service-delivery/package-of-interventions-for-rehabilitation) and other products in leadership and governance, financing, workforce, assistive technology, health information, and emergencies supports countries in strengthening rehabilitation in their health systems. On this background, the Rehabilitation 2030 initiative and its products can also serve as an enabler to strengthen health systems for LBP care. In particular, the close synergy between the WHO Guideline and the Package of Interventions for Rehabilitation for LBP creates a pathway for cooperation on implementation efforts relevant to LBP care*(5)*.

**References**

1. United Nations Department of Economic and Social Affairs, Population Division.,. World Population Prospects 2022. New York: Population Division, United Nations; 2024 (<https://population.un.org/wpp/Download/Standard/MostUsed/>, accessed 8 April 2024).

2. Ferreira ML, de Luca K, Haile LM, Steinmetz JD, Culbreth GT, Cross M et al. Global, regional, and national burden of low back pain, 1990-2020, its attributable risk factors, and projections to 2050: a systematic analysis of the Global Burden of Disease Study 2021. The Lancet Rheumatology. 2023;5:e316-e29. doi: 10.1016/S2665-9913(23)00098-X.

3. World Health Organization. UN Decade of Healthy Ageing: Plan of Action 2021-2030. Geneva: World Health Organization; 2020 (<https://www.who.int/publications/m/item/decade-of-healthy-ageing-plan-of-action>.

4. Cieza A, Causey K, Kamenov K, Hanson SW, Chatterji S, Vos T. Global estimates of the need for rehabilitation based on the Global Burden of Disease study 2019: a systematic analysis for the Global Burden of Disease Study 2019. Lancet. 2021;396:2006-17. doi: 10.1016/s0140-6736(20)32340-0.

5. World Health Organization. Package of interventions for rehabilitation: module 2: musculoskeletal conditions. Geneva: WHO; 2023 (<https://www.who.int/publications/i/item/9789240071100>.
