# Supplementary material for: The World Health Organization guideline for non-surgical management of chronic primary low back pain in adults: implications for equitable care and strengthening health systems globally
Source: Glob Health Res Policy. 2025 Jul 7;10:26. doi: 10.1186/s41256-025-00426-w (PMC12232859; doi:10.1186/s41256-025-00426-w)
Supplement: Supplementary file 3 — Additional file 3. [file 41256_2025_426_MOESM3_ESM.docx]

## WHO resources to support healthcare workers to deliver recommended interventions

- [Executive summaries](https://www.who.int/publications/i/item/9789240081789) of the Guideline in all six UN languages
- [Quick reference guide](https://cdn.who.int/media/docs/default-source/mca-documents/ageing/lbp/summary_who_guideline_lower-back-pain_brochure_v7.pdf?sfvrsn=80aec829_1) to support healthcare workers
- [Social medial assets](https://www.who.int/publications/i/item/9789240081789) for public health messaging about chronic primary LBP
- [WHO Science in 5: Low back pain](https://youtu.be/C1uRJXC19Bg?si=Ooi3z1gSfHHKL0pM)
- ICOPE technical resources, including [Guidelines on community-level interventions to manage declines in intrinsic capacity](https://www.who.int/publications/i/item/9789241550109); [ICOPE Handbook](https://iris.who.int/handle/10665/380175) (guidance for person-centred assessment and pathways in primary care); [ICOPE Handbook App](https://www.who.int/teams/maternal-newborn-child-adolescent-health-and-ageing/ageing-and-health/integrated-care-for-older-people-icope); [ICOPE implementation framework for systems and services](https://www.who.int/publications/i/item/9789241515993)
- [Package of Interventions for Rehabilitation](https://www.who.int/publications/i/item/9789240071100) of Musculoskeletal Conditions, including LBP
- [WHO Rehabilitation competency framework](https://iris.who.int/handle/10665/338782)
- [WHO UHC Compendium](https://www.who.int/universal-health-coverage/compendium) and the [UHC Service Planning, Delivery and Implementation](https://uhcc.who.int/uhcpackages/) (SDPI) platform
- [WHO mhGAP Intervention Guide (mhGAP-IG) for mental, neurological and substance use disorders in non-specialist health settings](https://www.who.int/publications/i/item/9789241549790) and [WHO mhGAP Evidence Resource Centre](https://www.who.int/teams/mental-health-and-substance-use/treatment-care/mental-health-gap-action-programme/evidence-centre)
- Aligned WHO Guidelines, including [WHO Guidelines on physical activity and sedentary behaviour](https://www.who.int/publications/i/item/9789240015128), [WHO Guidelines on the management of chronic pain in children](https://www.who.int/publications/i/item/9789240017870), [WHO Guidelines for the psychosocially assisted pharmacological treatment of opioid dependence](https://www.who.int/publications/i/item/9789241547543), and [WHO Guideline on the community management of opioid overdose](https://www.who.int/publications/i/item/9789241548816)
- [WHO Essential Medicines List](https://www.who.int/groups/expert-committee-on-selection-and-use-of-essential-medicines/essential-medicines-lists) (EML)
- [WHO Training in Assistive Products](https://www.who.int/teams/health-product-policy-and-standards/assistive-and-medical-technology/assistive-technology/training-in-products) (TAP) resource.
